# Safety and Performance of a New Burr Hole Covering Device: Results of the Multicenter COVER Registry

**DOI:** 10.1055/a-1883-0344

**Published:** 2023-05-23

**Authors:** Carlos Asencio-Cortés, Gloria Villalba, Álex De Vilalta, Laura Serrano, María Jesús Álvarez-Holzapfel, Guillermo Montes-Graciano, Xavier Málaga, Fernando Muñoz-Hernandez, Andreu Gabarrós

**Affiliations:** 1 Department of Neurosurgery, Hospital de la Santa Creu i Sant Pau, Barcelona, Spain; 2 Department of Neurosurgery, Hospital del Mar, Barcelona, Spain; 3 Department of Neurosurgery, Hospital Universitari de Bellvitge, L'Hospitalet de Llobregat, Barcelona, Spain; 4 Instituto Clavel, Hospital QuirónSalud Barcelona, Barcelona, Spain

**Keywords:** craniotomy, implant, polyether ether ketone, trephining

## Abstract

**Background** Burr hole covering in brain surgical procedures can avoid complications and unaesthetic results. The aim of this registry was to assess the safety and performance of a new polymeric burr hole covering device (Cranial COVER, NEOS Surgery).

**Methods** A multicenter, prospective, clinical registry design was used for the study. All the patients who fulfilled the inclusion criteria were included in the study and followed up for 6 months. Baseline clinical parameters, surgical variables (technical success of the implantation, surgeon satisfaction), postoperative variables (aesthetic and functional results, neuroimaging artifacts), and adverse events were evaluated.

**Results** Forty-three Cranial COVER devices were implanted in 30 patients. Most of them were implanted in frontal locations (53.5%). After implantation, 97.7% of the devices completely covered the burr hole, and 100% perfectly adapted to the skull surface. All surgeons ranked their satisfaction with the implantation procedure as very high or high. No artifacts were detected in any of the neuroimaging studies performed and no adverse events related with the device or its implantation were reported during the follow-up. There were significantly more scalp depressions associated with uncovered than with Cranial COVER–covered burr holes ( *p* = 0.040). Patient satisfaction with covered burr holes located in the frontal and parietal areas was 9.0 ± 1.4 over 10.

**Conclusion** Cranial COVER is a safe and reliable burr hole covering system that offers excellent cosmetic results and high satisfaction rates for both surgeons and patients. Cranial COVER is highly adaptable to the skull surface, and it was predominantly used in frontal locations due to their cosmetic importance.

## Introduction

Burr holes are the most common way to access the brain, either as stand-alone holes or as part of a craniotomy. Multiple systems have been used for the re-fixation of the bone flap after a craniotomy, but the covering of burr holes has been less considered throughout history. Considering their small size (the most usual perforators used to perform burr holes have an epicranial diameter of 14 mm and a subcranial diameter of 11 mm), frequently the burr holes are not specifically covered once the surgery is completed. This can lead to complications (herniation of intracranial structures cannot be completely ruled out, especially if there is an increase in intracranial pressure) 1 or to unsightly, unaesthetic depressions of the skin or the scalp in the area of the hole.

The first obvious solution to cover a bone defect, particularly if it is small (i.e., a burr hole), is to use bone itself. Some have proposed the possibility of using buttons made of bone autografts, obtained from the inner table of the bone flap, to guarantee that there is no reaction to foreign materials and make the end of the surgery easier. 2 This is in line with another common clinical practice: filling the burr holes with a pastry made of wet bone dust—harvested from the patient at the beginning of the procedure—to facilitate bone regeneration in the area. 3 4 This is an inexpensive method, easy to use, and can also offer a mostly positive cosmetic outcome. Furthermore, various adjuncts to the autologous bone dust have been described, for example, it can be augmented with fibrin glue 5 or previously compressed into a solid plug. 3 6 7 However, this method has been related to postoperative complications, particularly for stand-alone burr holes performed in endoscopic neurosurgery, caused by the migration of the material used to close the burr hole into the endoscopy tract. 8

The use of metal plates and screws, particularly made of titanium, in cranial surgery is also frequent. 9 Specifically, round-shaped plates of several diameters are available from different manufacturers to cover burr holes. These plates are screwed to the cranial bone surrounding the hole (either part of a craniotomy or isolated), with up to five or six screws, and have proved to be effective to reduce the aesthetic problems associated with burr holes. 10

In this study, we assess a conceptionally new product for covering burr holes: a polymeric clamplike device (Cranial COVER, NEOS Surgery, S.L., Barcelona, Spain). This clamplike device can be used both in isolated and craniotomy burr holes. This registry had the main aim of evaluating the clinical safety and performance of Cranial COVER as a burr hole cover in neurosurgical procedures in a “real-world” cohort of patients.

## Patients and Methods

### Study Design and Setting

This is an observational, prospective, single-arm, multicenter cohort study. Recruitment and follow-up took place between March 2019 and August 2020, at four tertiary centers in Spain. The study protocol was approved by the Ethics Committee of each hospital (References: HSCSP 18/325 (PS); GHQSB 53/2018; PSM 2019/8693/I; HUB PR389/18) and the investigation was conducted according to the principles and rules laid down in the Declaration of Helsinki and its subsequent amendments. This report follows the Strengthening the Reporting of Observational Studies in Epidemiology (STROBE) Statement: guidelines for reporting observational studies.

### Study Population

Patients were eligible for inclusion in the registry if they were >3 years of age and had to undergo a cranial surgery, where at least one burr hole was planned to be performed. Key exclusion criteria were fever or leukocytosis, allergy to implant material, degenerative bone disease, and use of artificial cranial bone. The eligibility of subjects was also assessed intraoperatively: the burr hole cover was implanted in patients with burr holes whose diameter was between 10 and 14 mm, and that did not have infection, inflammation, or bone tumors in the operated area. Patients with burr holes located in the sinus or in the inferior occipital cranial area, or without a suitable tissue cover in the operative field, were intraoperatively excluded from the study.

Patients included in this registry were followed up according to standard clinical practice of each study center for 6 months after surgery, until they were clinically discharged, lost to follow-up, or dead.

### Intervention: Device Description and Implantation Technique

The device evaluated in this clinical investigation was Cranial COVER (NEOS Surgery S.L), a CE-marked device. Cranial COVER is intended to cover burr holes, either stand-alone holes or holes being part of a craniotomy. Cranial COVER is totally made of the polymer polyether ether ketone (PEEK) and is based on the principle of a clamp: it consists of two platforms linked by two adjustable cable ties, and it is kept tightened to the skull with a double locking system in the upper platform. This is based on two ratchets that engage with the lower platform's cable ties' teeth that allow its movement toward the lower platform and, at the same time, impede its backward movement. The device is available in two sizes: the large size, for use in the most common burr holes, made with standard perforators of diameters 14/11 and 13/9 mm; and the small size, for use in burr holes with a diameter in the range of 10 to 12 mm made with high-speed spherical drills.

**Fig. 1:**
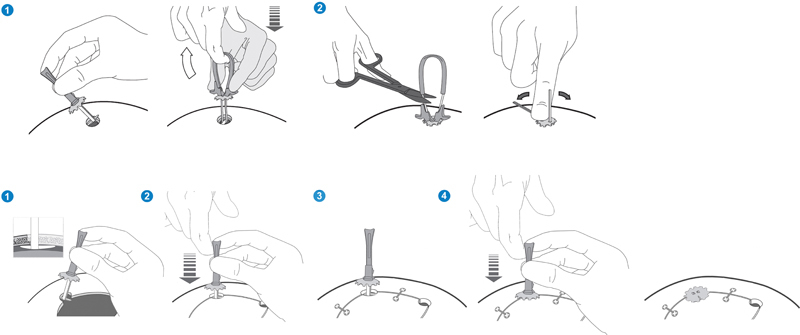
Top: Implantation technique of Cranial COVER in a single burr hole: (1) Cranial COVER's elongated lower platform is introduced through the burr hole and then the device is tightened by pulling the handle and adjusting the upper platform on the skull surface. (2) The cable ties are cut to remove the handle and the applier. Finally, the protruding excesses of the cable ties are cut and removed by bending them repeatedly up and down. Bottom: Implantation technique of Cranial COVER in craniotomy burr holes before repositioning the bone flap: (1) The lower platform is prepositioned in the burr hole area, between the bone and the dura mater. (2) The bone flap is placed back in the skull and the device is initially adjusted to prevent its movement. (3) The bone flap is fixed with specific products (e.g., plates). (4) The Cranial COVER is completely adjusted to the skull surface and the handle and applier are removed as described in the top approach.

**Fig. 2:**
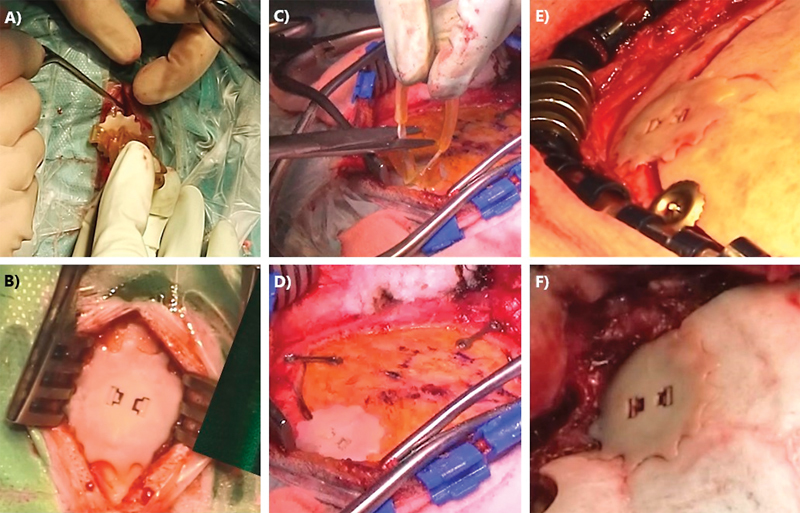
Implantation of Cranial COVER ( **A,B** ) in a single burr hole and ( **C,D** ) in craniotomy burr holes. Note that it can be used in combination with different craniotomy closure systems: ( **C,D** ) plates and screws and ( **E** ) clamps. ( **F** ) The device adapts to the skull surface, even in irregular areas, such as the pterion.

The implantation of Cranial COVER does not require any specific surgical instrument. It can be implanted following two different approaches:

- For stand-alone burr holes or for craniotomy burr holes where the bone flap has been previously repositioned in its original location: First, the Cranial COVER's elongated lower platform is introduced through the burr hole, into the subcranial area, between the bone and the dura mater and centered in the hole axis. Then, the device is tightened by pulling the handle and adjusting the upper platform on the burr hole surface by sliding it down with the help of the applier, until the upper platform is properly adapted to the skull surface. The upper platform then remains in its position thanks to its double locking system. Afterward, the cable ties are cut using standard instruments (e.g., Mayo scissors) to remove the handle and the applier. Finally, the protruding excesses of the cable ties are cut and removed taking advantage of their self-cutting feature, by bending them repeatedly up and down.
- For craniotomy burr holes where the bone flap has not been previously repositioned in its original location only: First, the lower platform is prepositioned in the burr hole area, between the bone and the dura mater, before positioning of the bone flap. Then, the bone flap is placed back and the device is initially adjusted to prevent its movement while the bone flap is fixed with specific products (e.g., plates). Finally, the Cranial COVER is completely adjusted to the skull surface, and the handle and applier are removed as described in the first approach ( Figs. 1 and 2 ).

### Outcomes

The primary endpoint of the study was to assess the prevalence of patients who have a complete covering of the cranial burr hole at the end of the surgery. Burr hole covering and adaptation was evaluated according to the following classification with four levels: <25% of the upper platform contacts the cranial surface; between 25 and 50% of the upper platform contacts the cranial surface; between 50 and 75% of the upper platform contacts the cranial surface; and >75% of the upper platform contacts the cranial surface.

Secondary outcomes comprised both safety endpoints (the prevalence of patients who present any device-related adverse event or device deficiencies) and performance endpoints. The latter included the surgeon's overall satisfaction with the procedure of covering the burr hole, assessed by a Likert scale (scoring 1–5, where 1 means “very dissatisfied” and 5 means “very satisfied”); the surgeon's evaluation of the presence of scalp depression, by direct visual control of the surgical area during follow-up visits, measured as a dichotomic variable (yes/no); the satisfaction of patients with the aesthetic result of covered and uncovered burr holes, assessed by a Visual Analog Scale (VAS score 1–10, where 1 means “totally unsatisfied” and 10 means “completely satisfied”); the prevalence of patients who presented discomfort or functional handicaps associated with the covered and uncovered burr holes; and the presence of artifacts generated by the implanted products on computed tomography (CT) or magnetic resonance (MR) imaging.

### Statistical Analysis

Descriptive statistics were used to characterize the population studied and the different variables evaluated in all time points: in the preoperative period, in the surgery visit and at different follow-up times. For continuous variables (e.g., age, weight, etc.), arithmetic mean, standard deviation, median, minimum, maximum, and interquartile ranges (IQR) are presented. Categorical variables (e.g., gender) are presented in relative and absolute frequencies. Comparison of covered and uncovered burr holes was performed by means of Fisher's exact test or Mann–Whitney *U* test when appropriate. A *p* -value <0.05 was considered statistically significant. GraphPad Prism 6.01 (La Jolla, California, United States) was used to perform the analysis.

For sample size calculation, the primary endpoint of the study was considered: the aim was to confirm that a complete and solid covering of the burr hole was achieved in 90% of the patients. Bearing in mind that this threshold can be considered both in terms of performance and of safety, it is reasonable that, for the sake of calculating the sample size, the value for adequate burr hole covering (90%) was transformed into the frequency of an adverse event. This corresponds, in this case, to considering that the frequency of incomplete and/or nonsolid covering of the burr hole was 10%. According to Hanley's formula, 11 *n* = 30 patients is the sample size needed to detect an adverse event that occurs in the following conditions: 95% of confidence interval of the probability of occurrence of the event ( *α* = 0.05) and 10% of probability of occurrence of the adverse event. To account for possible losses (10%) at follow-up (that may affect the evaluation of secondary objectives), this number was increased to reach *n* = 33 patients.

## Results

### Study Population, Demographics, and Baseline Clinical Characteristics

Thirty-three patients were screened for inclusion in the study. All of them fulfilled the inclusion/exclusion criteria. However, three patients were finally not included as written informed consent was not obtained. Of the 30 patients included, 19 patients completed the 6-month follow-up, 9 patients performed at least one follow-up visit, but the follow-up time was less than 6 months, and 2 patients were discontinued because of an adverse event (not related to the study product or procedures). Results from the analysis of these 30 patients are presented in the following sections. The main sociodemographic and baseline clinical characteristics of the population studied are presented in Table 1 .

**Table 1:** Demographic and clinical characteristics of patients included in the study

Age (y)	*N*	30
	Mean (SD)	53.17 (16.11)
	Median (Min–Max)	53.5 (26.00–84.00)
	IQR	39.5–68.25
Age (distribution), *n* (%)	<18 y	0 (0)
	18–65 y	22 (73.33)
	>65 y	8 (26.67)
Gender, *n* (%)	Female	19 (63.3)
	Male	11 (36.7)
Smoking status, *n* (%)	Yes	3 (10.0)
	No	25 (83.3)
	Unknown	2 (6.67)
Baseline associated pathology, *n* (%)	Glioma	10 (33.3)
	Meningioma	6 (20.0)
	Metastasis	1 (3.3)
	Melanoma	1 (3.3)
	Hematoma or hemorrhage	3 (10.0)
	Aneurysm	2 (6.7)
	Cavernoma	2 (6.7)
	Epilepsy	3 (10.0)
	Head trauma	1 (3.3)
	Unknown tumor	1 (3.3)
Brain surgery history	First surgery	26 (86.7)
	Second or subsequent surgery	4 (13.3)
BMI (kg/m ^2^ )	*N*	21
	Mean (SD)	25.05 (5.29)
	Median (Min–Max)	24.21 (17.12–38.20)
	IQR	22.07–26.39

Abbreviations: BMI, body mass index; IQR, interquartile range; Max, maximum; Min, minimum; SD, standard deviation.

### Surgery Data

A total of 82 burr holes were performed in the 30 patients, 43 of which were covered with Cranial COVER (52.4%), 5 were covered with titanium burr hole plates (6.2%), and 34 were left uncovered (41.5%). Twenty patients were implanted with one Cranial COVER, 7 patients were implanted with two, and 3 patients were implanted with three devices. Details about the surgical procedure, the number and the location of burr holes performed, and the covering used in each of them are provided in Table 2 .

**Table 2:** Surgery characteristics of patients included in the study

Surgical procedure, *n* (%)	Craniotomy	27 (90.0)
	Hemorrhage drainage	2 (6.7)
	Cranioplasty	1 (3.3)
Burr holes performed/patient, *n* (%)	**1**	**4 (13.3)**
	Cranial COVER	4 (100)
	Uncovered	0 (0)
	Titanium plates	0 (0)
	**2**	**5 (16.7)**
	Cranial COVER	9 (90)
	Uncovered	1 (10)
	Titanium plates	0 (0)
	**3**	**16 (53.3)**
	Cranial COVER	23 (48)
	Uncovered	20 (42)
	Titanium plates	5 (10)
	**4**	**5 (16.7)**
	Cranial COVER	7 (35)
	Uncovered	13 (65)
	Titanium plates	0 (0)
Perforator used, *n* , (%)	Standard single use perforator 14/11 mm	80 (97.6)
	Standard single use perforator 13/9 mm	2 (2.4)
Burr holes location, *n* , (%)	**Frontal**	**32 (39.0)**
	Cranial COVER	23 (71.9)
	Uncovered	7 (21.9)
	Titanium plates	2 (6.2)
	**Parietal**	**21 (25.6)**
	Cranial COVER	11 (52.4)
	Uncovered	8 (38.1)
	Titanium plates	2 (9.5)
	**Temporal**	**20 (24.4)**
	Cranial COVER	7 (35.0)
	Uncovered	13 (65.0)
	Titanium plates	0 (0)
	**Occipital**	**9 (11.0)**
	Cranial COVER	2 (22.2)
	Uncovered	6 (66.7)
	Titanium plates	1 (11.1)
Burr holes laterality, *n* (%)	Left	45 (54.9)
	Right	37 (45.1)
Covering type/ burr hole location, *n* (%)	**Cranial COVER**	
	Frontal	23 (53.5)
	Temporal	7 (16.3)
	Parietal	11 (25.6)
	Occipital	2 (4.7)
	**Uncovered**		
	Frontal	7 (20.6)
	Temporal	13 (38.2)
	Parietal	8 (23.5)
	Occipital	6 (17.7)
	**Titanium burr hole plate**		
	Frontal	2 (40.00)
	Temporal	0 (0.0)
	Parietal	2 (40.00)
	Occipital	1 (20.00)
Implantation procedure of Cranial COVER, *n* (%)	Prepositioning (before the bone flap positioning)	16 (37.2)
	After closing the craniotomy	23 (53.5)
	Unknown/not applicable (single burr hole implantation)	4 (9.3)
Bone thickness at the level of implantation (mm)	*N*	43
	Mean (SD)	9.3 (3.3)
	Median (Min–Max)	10.0 (3.8–17.0)
	IQR	7.0–11.40

Abbreviations: IQR, interquartile range; Max, maximum; Min, minimum; SD, standard deviation.

### Primary Outcome

The primary endpoint of the study was to assess the prevalence of patients who present a complete covering of the cranial burr hole at the end of the surgery.

After the surgery, 42 of 43 (97.7%) Cranial COVER devices completely covered the burr hole, and all devices (43) had their upper platform completely adapted to the skull surface.

### Secondary Outcomes

#### Surgeons' Evaluations

The surgeon overall satisfaction with the procedure of covering the burr hole with Cranial COVER was evaluated after surgery. Surgeons rated it as “satisfied” or “very satisfied” in all cases.

**Fig. 3:**
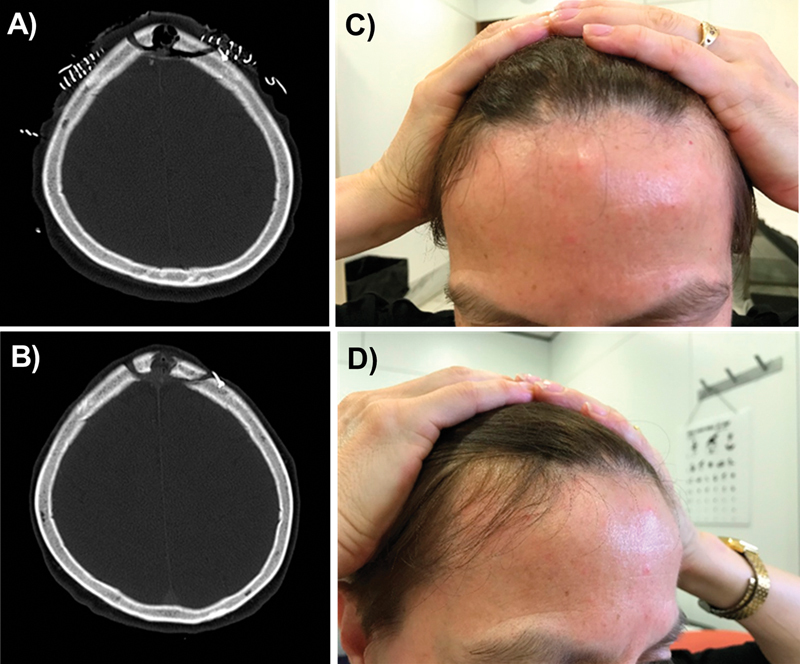
( **A** ) Postoperative and ( **B** ) follow-up (156 days postsurgery) computed tomography (CT) scan of a patient operated from a frontal left meningioma. ( **C,D** ) Aesthetic result in the follow-up visit. Note that there is no scalp depression in the burr hole area.

In the follow-up visit, the surgeons evaluated if there were any visible scalp depression, both in the area of implantation of Cranial COVER and in the area of uncovered burr holes. There were more statistically significant ( *p* = 0.04; Fisher's exact test) scalp depressions in those burr holes that were left uncovered (3, 21.4%) than in those burr holes that were covered with Cranial COVER (0, 0.0%; see Fig. 3 ). Similarly, no scalp depressions were observed in the burr holes covered with plates. However, in two out of the three patients with plates, a small bulge in the area of implantation was detected by the neurosurgeon.

#### Patient-Reported Outcomes

During follow-up visits, satisfaction with the aesthetic results of Cranial COVER was evaluated by 25 patients, while satisfaction with the aesthetic results of uncovered burr holes was evaluated by 14 of these patients (those who also presented these uncovered burr holes). Patient satisfaction score with covered burr holes was 8.9 ± 1.1, while it was 8.1 ± 1.7 with uncovered burr holes ( *p* = 0.11; Mann–Whitney *U* test). A subanalysis of frontal and parietal burr holes was then performed to assess those burr holes with a higher aesthetic impact. Patients reported a higher satisfaction score for parietal and temporal burr holes covered with Cranial COVER (9.0 ± 1.4) than for those left uncovered (7.8 ± 1.8; *p* = 0.06, Mann–Whitney *U* test). Differences were less pronounced in burr holes with a lower aesthetic impact (i.e., those located in temporal and occipital areas): patient satisfaction score with covered burr holes was 8.9 ± 0.6 in these locations, while it was 8.5 ± 1.4 with uncovered holes in these areas ( *p* = 0.85).

In follow-up visits, patients were also asked if they presented discomfort associated with the covering system and if they presented any functional handicaps during activities of daily living, such as hairdressing, combing, scratching the scalp, or washing their hair. Only three patients reported some kind of discomfort associated with the device implanted and one patient reported functional handicaps. Patients also reported a similar degree of discomfort and functional handicaps associated with uncovered burr holes ( *p* = 1.00; Fisher's exact test).

#### Radiologic Evaluation

The presence of artifacts in the postoperative CT or MR images was evaluated for each device implanted. No artifacts were detected in any of the images evaluated (see Fig. 3 ).

### Safety Assessment

No intraoperative or follow-up device-related adverse events or device deficiencies were reported in any of the studied patients.

Even though the presence of a blood clot was detected under three of the implanted devices in the postoperative images, it should be noted that all of them were reabsorbed and not present in the follow-up images. None of them was related with any adverse event.

Two serious adverse events (SAE) were reported during the study (two brain tumor recurrences). Neither of them was related to the product, but to the baseline pathology progression.

## Discussion

This study demonstrates that Cranial COVER is a safe and reliable burr hole covering system. A total of 82 burr holes were performed in the 30 patients included in the study in the four participating sites, 43 of whom were covered with Cranial COVER devices.

Cranial COVER was mainly implanted to cover burr holes performed in craniotomy or cranioplasty procedures (28 patients), but in 2 patients, it was used in single burr holes performed for hematoma drainages (three devices implanted in single burr holes). The fact that most of the devices were implanted in craniotomy burr holes reflects the daily clinical practice of most neurosurgery departments, where craniotomy procedures are much more frequent than those where single burr holes are performed. Moreover, two additional circumstances that limit the implantation of Cranial COVER in single burr holes should be considered: the first one is that Cranial COVER cannot be implanted in single burr holes where a drainage catheter is used and the second one is that, in some hospitals, small burr holes (smaller than the ones where Cranial COVER is indicated) are performed to treat subdural hematomas.

The implantation technique used to implant Cranial COVER in craniotomy procedures depended on surgeon preference, and both implantation techniques (before or after positioning the bone flap) were similarly used.

Regarding location, it is worth noting that Cranial COVER was mainly implanted in frontal and parietal locations, while temporal and occipital burr holes were predominantly left uncovered. Both situations point in the same direction: Cranial COVER devices are preferentially used in areas that have a higher aesthetic impact, as frontal and parietal areas are more prone to the presence of visible scalp defects due to, for example, the smaller presence of hair. Indeed, the fact that patients can potentially develop cosmetic complexes caused by the scalp depressions associated with burr holes is a frequent reason to cover these with a specific device, particularly in young patients and/or with good prognosis. The presence of thin skin in the area (which can be associated with several factors, including low body weight, advanced age, or treatment with radiotherapy, either before or after the surgical procedure) is another aspect that may lead to the use of burr hole covering devices. In fact, in these patients, to avoid the ulcerations that have been associated with plates before, 12 Cranial COVER may be considered particularly appropriate.

The primary study goal was to assess if Cranial COVER completely covers the burr hole: this was achieved with almost all of the Cranial COVER devices implanted, which proves the product's adequate performance. In addition, all 43 devices were completely adapted to the skull surface, thus further confirming the appropriate performance of the device. In fact, surgeons ended all surgical interventions satisfied or very satisfied with the implantation procedure of Cranial COVER.

Secondarily, the study also aimed to assess aesthetic results of the implant and self-perception patient evaluations.

Based on the surgeons' evaluation, there were significantly more skin concavities present over uncovered holes than over holes covered with Cranial COVER. However, patients ranked their satisfaction with covered and uncovered burr holes similarly. A potential explanation for this is that, as previously discussed, Cranial COVER was predominantly implanted in areas with a higher aesthetic impact (frontal and parietal), while uncovered burr holes were predominantly located in temporal and occipital regions, which have a smaller cosmetic importance; thus, their negative aesthetic impact may be considered less relevant. In fact, when analyzing only those burr holes performed in frontal and parietal locations, patients did give higher satisfaction scores when burr holes were covered with Cranial COVER than when burr holes were left uncovered.

We also confirmed that Cranial COVER does not generate any artifacts in the neuroimages (neither CT scan nor MRI).

From a safety point of view, it is relevant to note that neither intraoperative nor follow-up complications were registered in the study.

This is the first study that has prospectively and systematically evaluated the use of a burr hole covering system after a neurosurgical procedure, adding an important level of clinical evidence to the procedure of covering burr holes after cranial surgery, which has been seldom investigated so far. A previous pilot retrospective study evaluated a total of 14 burr hole covers (titanium plates) placed in 11 patients and compared them to 50 burr holes that were not covered. 13 Through telephone interviews with the patients, this study showed that patient satisfaction with the aesthetic outcome was significantly better for covered burr holes and that skin depressions were present in 7% of covered burr holes and 92% of uncovered burr holes. Similar results were also reported in another retrospective study on 196 burr holes (101 uncovered and 95 covered with a titanium plate) in 162 patients. In this case, based on the evaluation of the latest follow-up brain CT scan image available, the incidence of scalp depressions was 7.4% in the patients with covered burr holes and 91.1% in the patients with uncovered burr holes. 10

Although in our study the incidence of scalp depressions over uncovered burr holes was smaller, this can be explained by at least two factors: first, because the evaluation was performed visually by a neurosurgeon (as opposed to the patient evaluation in Vasella et al 13 and the radiologic evaluation—a depression on a CT scan image may not result in a depression visible from the outside—in Im et al 10 ) and, second, because uncovered burr holes were predominantly located in temporal and occipital regions, where the visual examination may be more difficult due to the presence of more hair. In addition, both studies were performed in patients with stand-alone burr holes, while our study includes mostly burr holes that are part of a craniotomy, and this factor may have some impact on the measured outcome. In any case, all studies conclude that burr hole trepanations may lead to delayed scalp depressions and an unsatisfactory aesthetic outcome that, as we have seen in our and in previous studies, can be prevented using a burr hole cover. Despite this, the use of burr hole covers is still not a standard clinical practice. 14

Studies published during the last few years have proved that the use of piezosurgery to perform craniotomies could avoid the existence of burr holes in aesthetically relevant areas. 15 16 However, this technique is not yet broadly implemented and some studies have indicated that surgery is significantly prolonged. 16 .

Our results show that this new polymeric burr hole covering device can completely prevent scalp depressions, similar to other cover systems, such as plates. Nonetheless, the new burr hole cover can have some advantages if compared with titanium plates: the low profile of the device avoids skin bulges; it is quickly and easily implanted (high surgeon satisfaction scores have been achieved in this study); it does not require any particular instrument for implantation (thus, it saves time in the operating room and does not need to be sterilized before use). Based on this, Cranial COVER is plausibly a cost-effective solution to cover burr holes.

It is worth noting that in a craniotomy fixed with the Cranial LOOP system, the XL size could be used for both covering and fixing the bone flap 17 ; however, Cranial COVER would be of use for an aesthetic cover of the burr hole in a craniotomy fixed with plates and screws.

Other not so large devices for covering burr holes are the so-called buttons. These usually consist of a small piece, in the shape of a mushroom and with a diameter that fits with the hole diameter, which is plugged into the hole at the end of the surgical procedure. Multiple materials have been proposed for these devices, including both absorbable (polycaprolactone) 18 19 and nonabsorbable (polyethylene) polymers, 20 and ceramics such as hydroxyapatite, 21 22 23 in all cases with satisfactory cosmetic outcomes.

Covering the burr hole with bone dust or polymethyl methacrylate (PMMA) is a less costly solution. However, after a variable period of time, cranial defects treated only with bone dust demonstrate local skin depressions, probably caused by a high level of bone resorption, which leads to unfavorable cosmetic results 24 . Iin some cases with both autologous bone dustand PMMA cement may lead to significant morbidity. 25 Studies that have measured the depth of scalp depressions associated with burr holes in the long term, using CT scan images, confirm this. The available results show that depression depth is 1.24 ± 0.78 mm in burr holes treated with bone dust plugs (at 12 months of follow-up), 3 while it reaches only 0.16 ± 0.57 mm in burr holes covered with plates (at the latest follow-up image available; the mean follow-up was 20.65 weeks). 10 Recently, a study has also investigated the use of acellular dermal matrix as a burr hole cover. Initial results seem promising regarding the aesthetic result of the surgery. 26

This clinical investigation has some limitations. First, the lack of a control group does not allow for a direct comparison of covered versus uncovered burr holes in similar conditions, thus limiting the interpretation of results. However, this study aimed at obtaining “real-world” clinical data, and it sought not to interfere with the usual clinical practice, or the neurosurgeons' decision to cover burr holes with an implant, or in the scheduled follow-up visits.

Also, the study aimed at collecting 6 months of follow-up data, but this follow-up time was not reached in 11 patients, due to several reasons (adverse event, clinical discharge, or next follow-up visit scheduled after the end of the study). Again, the design of the study to collect real-world clinical data and to minimize the interference with daily clinical practice explains this situation: a fixed scheduled timing for follow-up visits was not established. Future studies including longer follow-up times and a larger cohort could also be of interest to confirm the safety and efficacy of the device in the long term.

## Conclusion

In summary, the results of this clinical registry prove that Cranial COVER is a safe device that completely covers both stand-alone and craniotomy burr holes, perfectly adapting to the skull surface. Furthermore, the use of Cranial COVER could have a positive aesthetic impact on patients, particularly in frontal and parietal burr hole locations, where cosmetic defects are more disturbing. Finally, it has been confirmed that Cranial COVER does not interfere with follow-up imaging, as it does not generate any artifact in CT and MRI.
